# Bibliographical

**Published:** 1869-11

**Authors:** 


### Editorial.

### BIBLIOGRAPHICAL.

*A Treatise on the Disease and Surgery of the Mouth und Jaws and Associate Parts.*

BY JAMES E GARRETSON, M. D., D. D. S.

We have received and examined to some extent this work, just issued from the press of D. Lippincott & Co. From the cursory examination we have made of the work, we must express a very high degree of satisfaction with the manner in which Professor Garretson has performed his work. From his reputation and known ability, however, a work of less merit would hardly be expected, though Dr. G. has certainly fulfilled the expectations and desires of the profession. It embraces a large range of observation and practical experiment, such as no other man, in this country at least, has enjoyed. It bridges over most completely the chasm heretofore so definitely marked between general and oral surgery.

The tendency of this book, wherever read, will be to elevate Dental surgery—to give to the Dentist an extended view of the domain of his operations. It is one of the very few works in our profession adapted to both student and practitioner. It will also be of great value to the surgeon and physician. It at once comes into the list of text books for colleges, and should have a place in the library of every Dentist, surgeon and physician. We commend the work because of its great value, and would not speak condemnatory because a few errors or lapses have crept in unawares. There is, perhaps, as few of these as was ever found in the first edition of so extensive and laborious a work.

For this work and labor of love, the profession owe Dr. Garretson a debt of gratitude that we trust they will not be slow to pay, so far as appreciation can do it.

We have not given a synopsis of its contents, feeling quite confident that every one who cares to know anything about it will procure the book, then enjoy it in all its fullness and richness. It may be had of any dealer in medical books. T.

